# Isolation, Culture, and Characterization of Chicken Cartilage Stem/Progenitor Cells

**DOI:** 10.1155/2015/586290

**Published:** 2015-08-16

**Authors:** Lu Li, Yuehui Ma, Xianglong Li, Xiangchen Li, Chunyu Bai, Meng Ji, Shuang Zhang, Weijun Guan, Junjie Li

**Affiliations:** ^1^College of Animal Science and Technology, Agricultural University of Hebei, Baoding 071000, China; ^2^Institute of Animal Science, Chinese Academy of Agricultural Sciences, Beijing 100193, China; ^3^Hebei Normal University of Science & Technology, Qinhuangdao 066000, China; ^4^Research Center of Cattle and Sheep Embryo Engineering Technique of Hebei, Baoding 071000, China

## Abstract

A chondrocyte progenitor population isolated from the surface zone of articular cartilage has become a promising cell source for cell-based cartilage repair. The cartilage-derived stem/progenitor cells are multipotent stem cells, which can differentiate into three cell types in vitro including adipocytes, osteoblasts, and chondrocytes. Much work has been done on cartilage stem/progenitor cells (CSPCs) from people, horses, and cattle, but the relatively little literature has been published about these cells in chickens. In our work, CSPCs were isolated from chicken embryos in incubated eggs for 20 days. In order to inquire into the biological characteristics of chicken CSPCs, immunofluorescence, reverse transcription-polymerase chain reaction (RT-PCR), and flow cytometry were adopted to detect the characteristic surface markers of CSPCs. Primary CSPCs were subcultured to passage 22 and, for purpose of knowing the change of cell numbers, we drew the growth curves. Isolated CSPCs were induced to adipocytes, osteoblasts, and chondrocytes. Our results suggest that we have identified and characterised a novel cartilage progenitor population resident in chicken articular cartilage and CSPCs isolated from chickens possess similar biological characteristics to those from other species, which will greatly benefit future cell-based cartilage repair therapies.

## 1. Introduction

The articular cartilage is physically self-repaired without vascular tissue, which consists of cartilage cells. It is the major bearing surfaces of joint. Injury to cartilage often progresses spatiotemporally from the articular surface to the subchondral bone, leading to the development of degenerative joint diseases such as osteoarthritis (OA) [1]. OA is characterized by progressive loss of articular cartilage, subchondral bone sclerosis, osteophyte formation, and synovial inflammation; clinical symptoms include activity limitation and pain [2]. OA is the most common cause of mobility loss, severely affects quality of life, work productivity, and cost of health care, and is the most prevalent form of musculoskeletal disease worldwide [3–5]. Due to the ability to form multiple tissue types, stem cells became the important material source for tissue regeneration, especially for the repair of degenerated tissues, including articular cartilage. In 1976, Thorogood PV and Hall BK made use of variable lactate/malic dehydrogenase ratios to distinguish between progenitor cells of cartilage and bone in the embryonic chick [6]. Alsalameh et al. firstly reported the identification of mesenchymal progenitor cells in normal and osteoarthritic human articular cartilage in 2004 [7]. Since then several independent research teams began to report that human cartilage stem/progenitor cells can be isolated [8–11]. Worthley et al. reported in 2014 that bone and cartilage could develop from a population of dedicated and committed postnatal progenitors (as with pancreatic beta cells). Alternatively, they could arise from a multipotent stem cell capable of generating bone, cartilage, and accessory elements, such as adipocytes and pericytes [12]. At present, more and more researchers believed that the articular cartilage stem/progenitor cells existed in order to maintain a steady state within the organization. But different research groups had different reports. Firstly, the source of CSPCs was not the same. Secondly, the methods of isolation and identification were different. So a lot of information was difficult to compare and reference. Thirdly, different groups used different genes to mark CSPCs.

Due to the fact that the chicken is an animal model that can provide abundant stem cells [13], we isolated CSPCs from the articular cartilage tissues of chicken embryos in incubated eggs for 20 days and cultured them in vitro. These cells were identified by expression of specific surface markers, thus tested for their ability to self-renew and differentiate.

## 2. Materials and Methods

### 2.1. Experimental Materials

Fertilized eggs were provided by the chicken breeding farm of the Chinese Academy of Agriculture Science, Beijing, China. All chickens were treated in accordance with the protocols and guidelines for agricultural animal research imposed by the Committee for Ethics of Beijing, China.

### 2.2. Isolation and Culture of CSPCs

The chicken embryos were obtained under sterile conditions. We removed the tibia and carefully scraped off the soft tissue and periosteum. Eye scissors were used to obtain either side of the distal tibia; proximal and articular cartilages were cleaned by washing them several times with phosphate buffer solution (PBS) without calcium or magnesium. Then cut up isolated cartilage. The tissues were chosen without vascular invasion and calcification. Chicken chondrocytes were isolated by a sequential 700 IU/mL pronase and 300 IU/mL collagenase digestion, as described previously [14]. The suspension was filtered through 74 *μ*m mesh sieve and centrifuged at 200 ×g for 10 min at room temperature. The supernatant was discarded and the pellet resuspended with medium containing DMEM/F12 and 10% (v/v) FBS.

With the aim of isolating a progenitor population, a differential adhesion onto fibronectin was performed [15]. Six-well culture plates were pretreated with 1% fibronectin solution diluted in sterile PBS plus 1 mM CaCl_2_ and 1 mM MgCl_2_ and incubated overnight at 4°C. Chondrocytes were seeded at 4000 cells/mL and incubated for 20 min in serum free media. After 20 min, the media plus nonadherent cells were carefully removed and replaced with medium (DMEM/F12 and 10% (v/v) FBS). The medium was firstly changed after 48h and then once per 2 days. After about 5 days, the confluence of cells reached 80%; the cells were labelled as P0 generation and then passaged every 2 days.

### 2.3. Growth Kinetics

Cells from passages 5, 10, and 15 were used to analyze growth kinetics of CSPCs. The cells were harvested and plated in 24-well microplates at a density of 10^4^ cells/well. After culture for 7 days, the cells from three wells were counted randomly each day and each well was repeated for 3 times. The population doubling time (PDT) was calculated based on the formula PDT = (*t* − *t*_0_) lg 2/(lg *N*_*t*_ − lg *N*_0_); *t*_0_ is starting time of culture; *t* is termination time of culture; *N*_*t*_ is ultimate cell number of culture; *N*_0_ is initial cell number of culture.

### 2.4. Karyotype Analysis

Chromosomes were prepared, fixed, and stained following standard methods [16]. After Giemsa staining, the chromosome numbers per spread were counted for 100 metaphase spreads under an oil immersion objective. Relative length, arm ratio, and centromeric index were calculated according to the protocol of Sun et al. [17] and Kawarai et al. [18].

### 2.5. Immunocytochemistry

Monolayer cultures of chicken cartilage stem/progenitor cells were fixed in 4% paraformaldehyde for 20 min and then washed three times in PBS (5 min each). The cells were permeabilized with 0.25% Triton X-100 for 15 min and washed three times in PBS (5 min each). The cells were blocked with 10% normal goat serum for 30 min and then incubated in PBS containing the following polyclonal antibodies, respectively: rabbit anti-collagen type II (1 : 100), rabbit anti-collagen type I (1 : 100), rabbit anti-aggrecan 1 (1 : 100), rabbit anti-SOX9 (1 : 100), rabbit anti-vimentin (1 : 100), and rabbit anti-FGFR3 (1 : 100) over night at 4°C. Next day, the cells were washed thoroughly with PBS three times for 5 min each time and incubated in PBS containing IgG. The cells finally were counterstained with DAPI. Photomicrographs were taken using Nikon TE-2000-E confocal microscope with an attached Nikon ZE-1-C13.70 digital camera system then quantified by a video densitometric analysis using image software.

### 2.6. RT-PCR Assay

RNA that was extracted using Trizol reagent [19] from passage five cells was used as a template for cDNA synthesis using Moloney Murine Leukemia Virus (M-MLV, Takara) according to the manufacturer's instructions. The primers were designed by primer 6.0 and were described in Table 1. RT-PCR was continued for 35 cycles after an initial denaturation at 94°C for 10 min. Each cycle of PCR consisted of 94°C for 30 sec, annealing temperature for 30 sec and 72°C for 30 sec, and a final extension for 10 min at 72°C. PCR productions were visualized on a 2.5% agarose gel with ethidium bromide [20].

### 2.7. Flow Cytometry

Cartilage stem/progenitor cells were counted and collected (passage 7) in logarithmic phase with conventional method. 1–5 × 10^5^ cells/pipe were transferred into the flow tube and then centrifuged at 1200 r/min for 8 min. After centrifugation the supernatant was discarded and cells were washed in PBS twice. Add the precooling 70% ethanol to the tube and gently blow into single cell suspension. Incubate them overnight at 4°C. The next day, they were centrifuged at 1200 r/min for 8 min to collect cells and washed in PBS once. The cells were permeabilized with 0.25% Triton X-100 for 15 min and washed in PBS twice. And then cells were incubated in PBS containing the following polyclonal antibodies, respectively: rabbit anti-collagen type II (1 : 100), rabbit anti-collagen type I (1 : 100), rabbit anti-aggrecan 1 (1 : 100), rabbit anti-SOX9 (1 : 100), rabbit anti-vimentin (1 : 100), and rabbit anti-FGFR3 (1 : 100) over night at 4°C. Next day, they were washed in PBS twice and incubated in PBS containing IgG. Finally, they were washed in PBS twice and subjected to single channel fluorescently activated cell sorting (FACS) analysis.

### 2.8. Cell Differentiation Assays

Cells from passage 5 were used to do differentiation experiment. Upon reaching 70–80% confluence, the whole cells were divided into three groups. Cells in the induced group one were transferred to osteogenic medium that consisted of DMEM/F12, 10% FBS, 10 mM *β*-glycerophosphate, 10 nM dexamethasone, and 0.1 mM L-ascorbic-acid-2-phosphate. The medium was changed every 2 days. Three weeks later, the capacity of the cells for calcium node formation was determined by alizarin red staining, and osteoblast specific genes were detected further using RT-PCR. The induced group two were transferred to adipogenic medium that was made up of DMEM/F12 with 10% FBS, 1 mM dexamethasone, 200 uM indomethacin, 0.5 mM IBMX, and 10 uM insulin. After 2 weeks, intracellular lipid accumulation was determined by staining with oil red O and adipocyte specific genes were detected further using RT-PCR. The induced group three were transferred to chondrogenic medium that included DMEM/F12 supplemented with 5% FBS, 1% ITS, 50 ug/mL L-proline, 0.1 um dexamethasone, 0.9 mM sodium pyruvate, 50 ug/mL L-ascorbic acid, and 10 ng/mL TGF-*β*3. Cells were incubated at 37°C and 5% CO_2_ and media changed every 3 days. After 3 weeks, identified by staining with alcian blue and chondrocyte, specific genes were detected further using RT-PCR, compared with those induced before.

## 3. Results

### 3.1. Isolation, Culture, and Morphology of CSPCs

**Figure 1:**
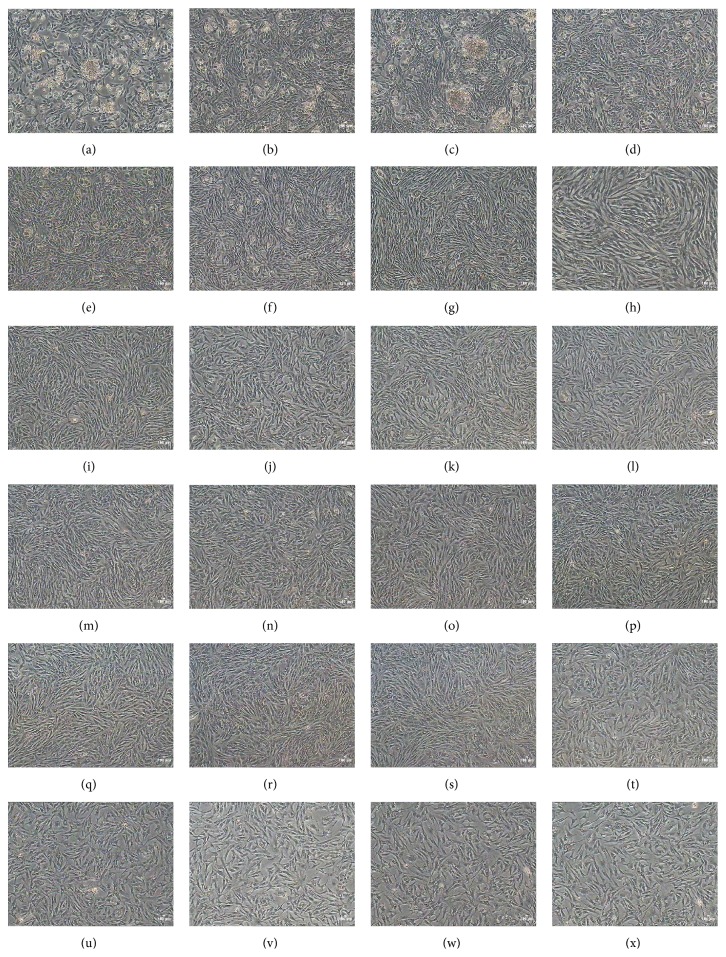
Cell morphology at different stages along with chicken CSPCs culture. (a) Chicken CSPCs on day 1 of primary culture, CSPCs cultured with some cartilage cells; (b) on day 3 of primary culture, the number of CSPCs increased and part of cartilage cells died; (c) CSPCs at P1. Cells increased quickly and cells aggregated in certain areas. (d) to (x) CSPCs at P2 to P22. During this time, cartilage cells died progressively and cells at P5 and P17 were homogeneous with a typical long spindle-shape. CSPCs at P18 appeared senescent.

After digestion for 20 min, the individual cells of cartilage inoculated to plate coated by fibronectin; some cells began to stick to wall. In the latter period of culture, cells grow as a colony. One week later, cells formed the large colony and the cells between colony and colony mutual confluence presented short spindle. After primitive culture, cells grew rapidly and can be passaged every 1 or 2 days. The cells were cultured to passage 5; the morphology of chicken CSPCs was uniform which displayed long fusiform or polygon. The cells cultured to passage 18 displayed a senescent appearance such as blebbing and karyopyknosis in most cells. Eventually, as passage numbers increased, the cells became detached from the plates (Figure 1).

### 3.2. Growth Kinetics

**Figure 2:**
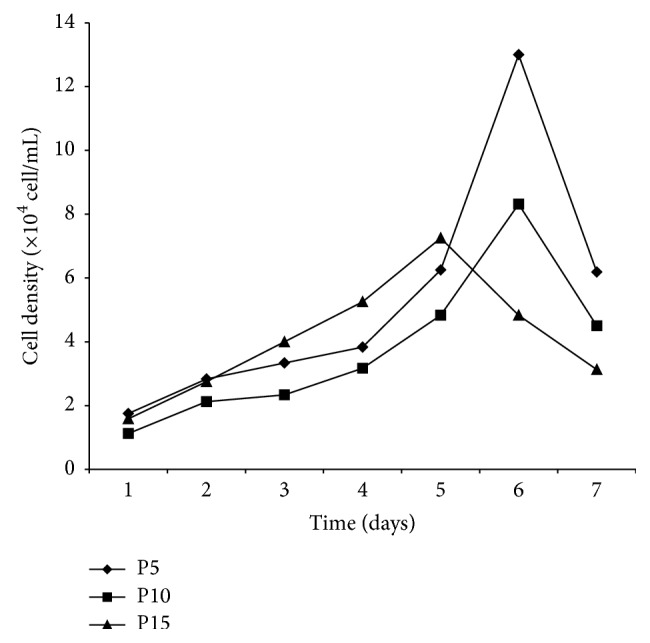
Growth curves of chicken CSPCs. The growth curves of P5, P10, and P15 CSPCs were all typically sigmoidal, with cell density reflected by the vertical axis. The growth curve consisted of a latent phase, a logarithmic phase, and a plateau phase. PDT calculated from the growth curve was approximately 47.98 h (*n* = 9).

The growth kinetics of CSPCs from different passages was shown by the growth curves. CSPCs entered the logarithmic phase after about 2.5 days and ended at sixth day. With increasing passages, the ability of cells to grow reduced (Figure 2).

### 3.3. Karyotype Analysis

**Figure 3:**
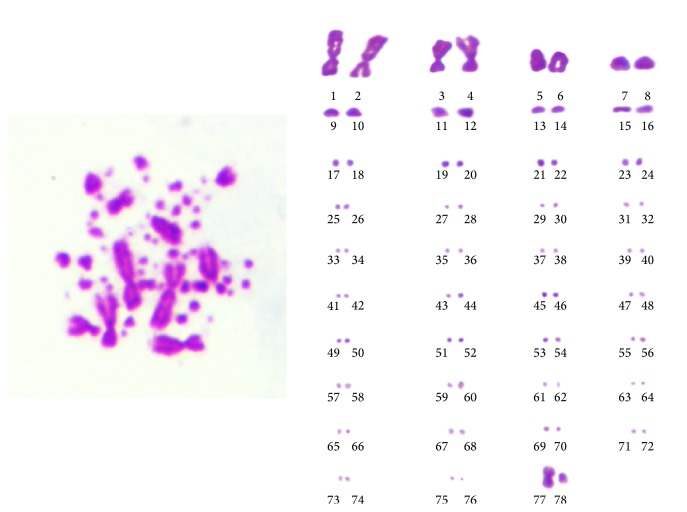
Karyotype of chicken CSPCs (♀) ZW type. The diploid chromosome number of chicken CSPCs was 2*n* = 78±, consisting of 9 pairs of macrochromosomes and 30 pairs of microchromosomes, with sex chromosome type ZZ (♂)/ZW (♀). A representative ZW (♀) type spread in metaphase was shown (*n* = 100).

The diploid chromosome number of chicken CSPCs was 2*n* = 78, consisting of 9 pairs of macrochromosomes and 30 pairs of microchromosomes, with the sex chromosome type being ZZ (♂)/ZW (♀). Chromosomal karyotype of chicken CSPCs was shown in Figure 3.

### 3.4. Immunofluorescence

**Figure 4:**
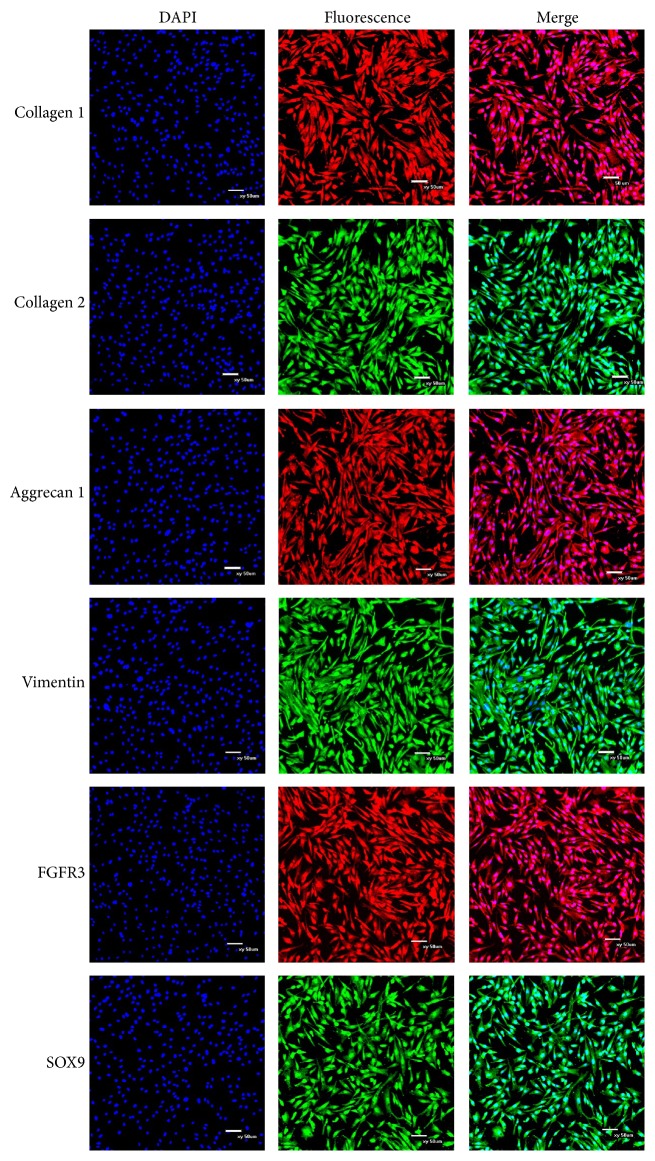
Immunolocalization of surface makers in CSPCs. Nuclei stained with DAPI are shown in the left panels. The pictures above indicated that markers of the chondrogenic phenotype, collagen 1, collagen 2, aggrecan 1, vimentin, FGFR3, and SOX9 were positive. The merged images are shown in the right panels. Scale bar = 100 *μ*m.

Specific marker proteins for CSPCs were detected through immunofluorescence staining. Expressions of collagen I, collagen II, aggrecan 1, vimentin, FGFR3, and SOX9 were observed in the CSPCs (Figure 4).

### 3.5. RT-PCR Analysis

**Figure 5:**
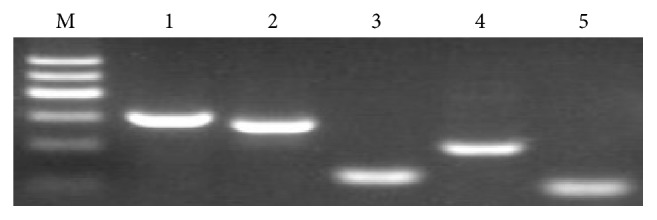
Detection of cell type specific makers via RT-PCR. The picture above showed that the expressions of GADPH, COL2A1, VIM, SOX9, and ACAN were all positive. Lane 1: GADPH was as internal control. Lane 2:COL2A1; lane 3: VIM;lane 4: SOX9; lane 5: ACAN. M: maker 600 bp.

RT-PCR experiments showed that the CSPCs expressed the relevant cell marker, COL2A1, VIM, SOX9, and ACAN. All the cell markers are positive (Figure 5).

### 3.6. FACS Analysis

**Figure 6:**
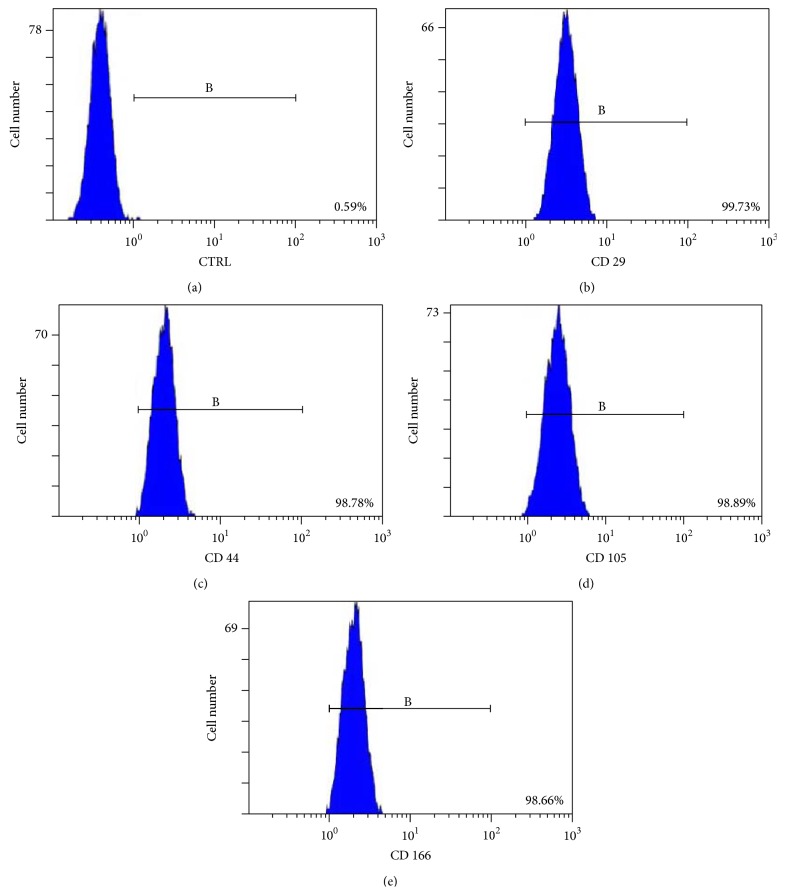
Flow cytometric analysis of CSPCs. CSPCs were labelled for MSC surface makers CD 29 (b), CD 44 (c), CD 105 (d), and CD 166 (e) that were reported in recent years. The control sample was sample (a). The positive rates of cells were all above 98%.

Fluorescently activated cell sorting (FACS) analysis was utilized to label CSPCs for a series of putative cartilage stem/progenitor cells markers. Analyses of CSPCs for CD29, CD44, CD105, and CD166 all showed expression over 98% of the viable cell population (Figures 1(b)–1(f)). However, expression of control was observed in a distinct population of 0.59%, within the viable CSPCs population (Figure 6).

### 3.7. Differentiation of CSPCs

#### 3.7.1. Adipogenic Differentiation of the CSPCs

**Figure 7:**
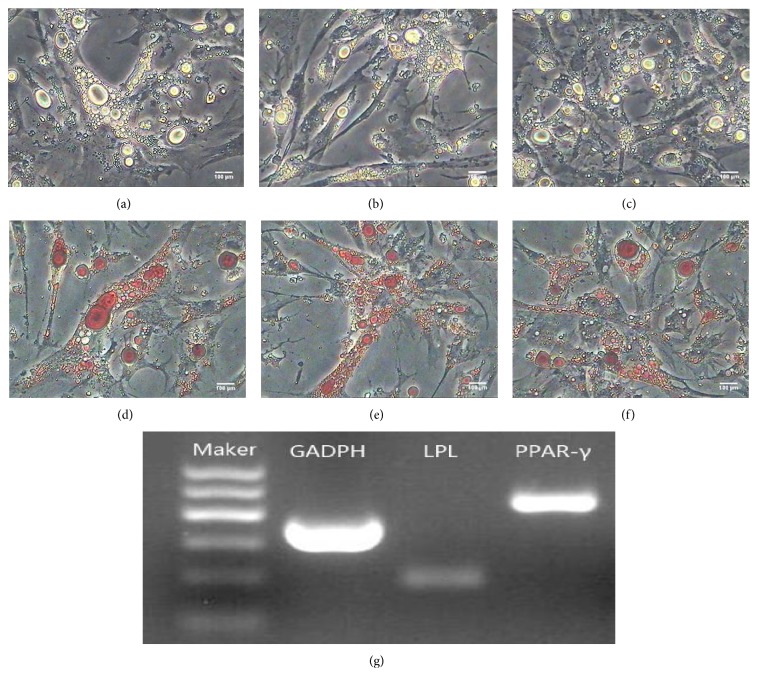
Adipogenic differentiation of chicken CSPCs. (a)–(c) After 2 weeks of induction, CSPCs metamorphosed from fibroblast-like shapes to oblate shapes and formed many lipid droplets in cells. Along with the prolongation of inducing time, droplets increased and aggregated to form larger ones gradually. (d)–(f) Lipid droplets displayedred through oil red O staining. (g) The expressions of adipocyte specific genes, including LPL and PPAR-*γ*, were detected by RT-PCR in the induced group after incubation for 2 weeks and these genes were all positive.

Adipogenic differentiation of the CSPCs was demonstrated by positive oil red O staining [21]. After incubation in adipogenic medium for 2 weeks, the CSPCs changed morphology from fibroblast-like to oblate, and many lipid droplets accumulated in the cells. The number of droplets increased in a time-dependent manner and tiny lipid droplets aggregated to form larger ones (Figures 7(a)–7(c)). After two weeks, cells were stained by oil red O (Figures 7(d)–7(f)). RT-PCR assay indicated that, after incubation with IBMX, insulin, and dexamethasone, the adipocyte specific genes, lipoprotein lipase (LPL), and peroxisome proliferator-activated receptor *γ* (PPAR-*γ*) were present (Figure 7(g)).

#### 3.7.2. Osteogenic Differentiation of CSPCs

**Figure 8:**
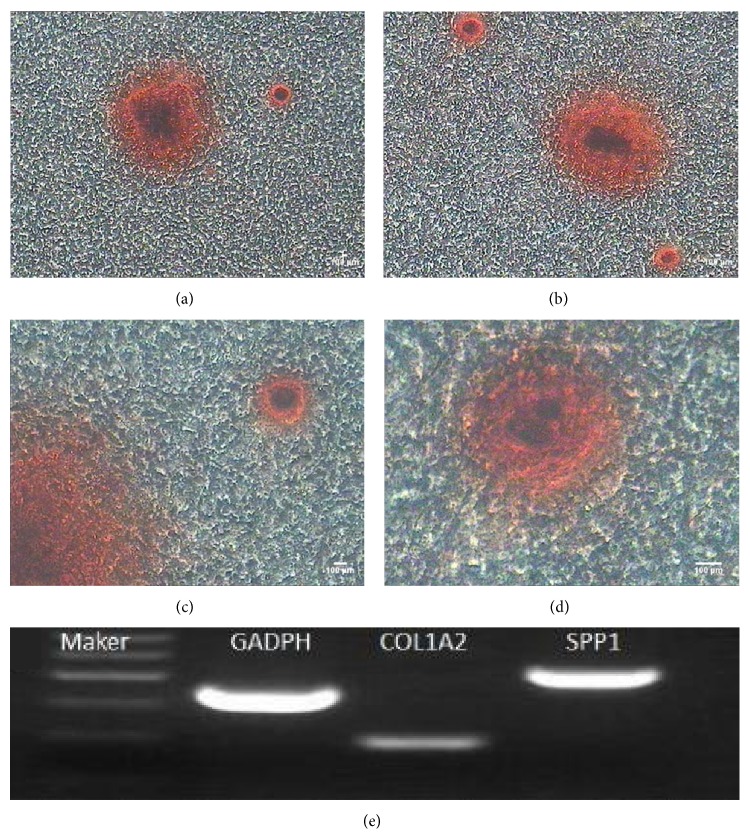
Osteogenic differentiation of CSPCs. (a)–(d) Following induction, the number of cells increased quickly and the nodules increased in number and became larger with prolonged induction. About 3 weeks later, nodules were observed after alizarin red staining. (e) RT-PCR assays revealed expression of osteoblast specific genes, including collagen type I, secreted phosphoprotein 1, at day 21 after induction.

**Figure 9:**
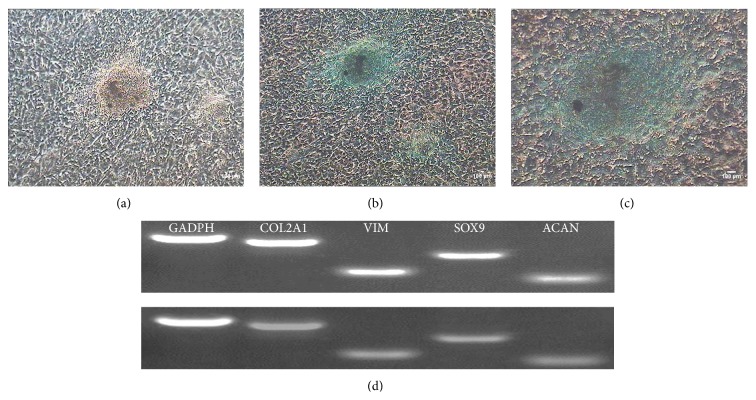
Chondrogenesis differentiation of CSPCs. (a) After incubation in chondrogenic medium for 1 month, the cells formed primmorphs that looked like cartilage. (b, c) Primmorphs were observed after alcian blue staining. (d) The comparison of PCR assays. The above one was detected before being induced and the below one was detected after being induced. Obviously, the expression of corresponding genes receded after being induced.

After incubation in osteogenic medium for 3 weeks, morphological changes in the CSPCs were obvious. The cells displayed significant changes in number. The nodules were stained with alizarin red (Figures 8(a) and 8(b)). As a result of the continued effects of the inducers, the nodules increased in number and size (Figure 9). Osteogenic differentiation of the CSPCs was analyzed by RT-PCR assay. The specific genes including collagen type I (COL1A2) and secreted phosphoprotein 1 (SPP1) were detected in the induced group (Figure 8(e)).

#### 3.7.3. Chondrogenic Differentiation of CMPCs

Chicken CSPCs proliferated after 7 days; some regions formed cluster-like aggregation. Proliferation continued with a relatively low speed. Meanwhile, the cellular configuration expanded, and the nuclear to cytoplasm ratio got higher. After incubation in chondrogenesis medium for 1 month, cells were detected with alcian blue. The primmorphs were stained as we can see. Chondrogenic differentiation of the CSPCs was analyzed by RT-PCR assay. The specific genes including COL2A1, VIM, SOX9, and ACAN tested before were all detected after being induced. Clearly, the bands were distinct.

## 4. Discussion

CSPCs were the main cell type used in cell-based cartilage repair therapies. The isolation and characterisation of CSPCs may offer a more favourable way for cartilage repair. In this paper, chicken CSPCs were isolated and partially characterised and the differentiation potential was shown. Previous research has relied on cell surface markers, originally designated to characterise bone marrow stromal cells, as a means of isolating possible stem cell populations from articular cartilage [7, 22, 23]. In a clinical situation, isolation of the correct cell population in suitable numbers would be dependent on an extremely consistent method of cartilage digestion and culture conditions, as these parameters play a significant role in experiment.

In light of these studies, we isolated a cartilage progenitor population with the fibronectin adhesion assay and selecting for cells that could then be expanded to high numbers [24]. We have demonstrated that CSPCs express the cells markers such as collagen I, collagen II, and some relevant makers using immunofluorescence and RT-PCR. Cell surface markers of the CSPCs always expressed CD29, CD44, CD166, and CD105, too. We detected their expression in chicken CSPCs with FACS analysis. CD29 is an integrin subunit, which is associated with later stage antigen receptors. It is essential for cell adhesion and recognition during embryogenesis, tissue repair, hemostasis, metastasis of malignant cells, and immune response [25]. CD44 is a receptor for hyaluronic acid and always can interact with other ligands including osteopontin, collagens, and matrix metalloproteinases. The function of CD44 is controlled by its posttranslational modifications. This protein participates in a wide variety of cellular functions including lymphocyte activation, recirculation and homing, hematopoiesis, and tumor metastasis [26]. In particular, research suggested that CD105 and CD166 were possible candidates of cartilage stem cell markers and they were the main makers of MSC.

Although bone itself possesses regenerative ability, the capacity for regeneration in other skeletal tissues (e.g., cartilage) is very low [27]. As stem cells can form multiple tissue types with their multipotential differentiation, they become a promising candidate cell type for tissue regeneration, particularly for the repair of degenerated tissues, including articular cartilage. As the new progenitor cells found in the articular surface, CSPCs can serve as ideal experimental cells for tissue engineering research. In our experiment, CSPCs were induced into osteoblasts, adipocytes, and chondrocytes in vitro and detected genes related to these cell types. We demonstrated that different inducing factors could affect the direction of differentiation of the CSPCS. The autologous nature of these stem cells, together with their putative multipotentiality and convenient procurement, makes them an excellent option for future tissue engineering and cell-based therapies [28]. The CSPCs, which as yet have no definitive marker, are responsible for the appositional growth of the tissue and exhibit a significant degree of plasticity in its differentiation pathway [15]. The existence of a progenitor population within the surface zone of articular cartilage opens up the possibility of using this population to engineer cartilage in vitro. Because these cells are undifferentiated, they should have the capability to reproduce the structural and hence biomechanical properties of normal articular cartilage and thus integrate more fully into articular cartilage lesions.

Although the differentiation of CSPCs was successfully identified in vitro, there are many technical difficulties and safety concerns related to using these cells for tissue recovery in vivo. Therefore, additional studies are needed with regard to using these cells for future research and therapy.

## 5. Conclusion

CSPCs were isolated from articular cartilage from 20-day-old chicken embryos. The self-renewal ability and differentiation potential of the isolated CSPCs were evaluated in vitro. Our findings provide the existence of CSPCs in chicken articular cartilage. Our findings always provide a platform for the establishment of a chicken CSPCs bank.

## Figures and Tables

**Table 1:** Primer sequences used in RT-PCR assay.

Gene name	Primer sequences	Product length (bp)	Tm (°C)
GADPH	F: 5′ CACAGAAGACGGTGGATG 3′	314	50
	R: 5′ ACCATTGAAGTCACAGGAG 3′		

COL2A1	F: 5′ GCGGAGATTACTGGATTGA 3′	293	50
	R: 5′ GTGGTAGGTGACGTTCTG 3′		

VIM	F: 5′ GCGTTGCTGCTAAGAATC 3′	141	50
	R: 5′ ACTGAATCTGTCTGCGATAT 3′		

SOX 9	F: 5′ GAGGCTGCTGAATGAGAG 3′	227	50
	R: 5′ TGATGCTGGAGGATGACT 3′		

ACAN	F: 5′ ATGCCGTTATTGCCACTC 3′	110	60
	R: 5′ TACACATCGTAGGTCTCTGA 3′		

PPAR-*γ*	F: 5′ CTGTCTGCGATGGATGAT 3′	199	47.3
	R: 5′ AATAGGGAGGAGAAGGAG 3′		

LPL	F: 5′ AGTGAAGTCAGGCGAAAC 3′	477	48.7
	R: 5′ ACAAGGCACCACGATT 3′		

COL1A2	F: 5′ AATGGTGGCGGATATGAAG 3′	190	50
	R: 5′ GGTGGCTAAGTCTGAGGT 3′		

SSPP1	F: 5′ CACTTCAGCAGACTCATTAC 3′	411	50
	R: 5′ TCTCTACGCTCTGATGTTG 3′		
